# Rapid and Direct Action of Lipopolysaccharide (LPS) on Skeletal Muscle of Larval *Drosophila*

**DOI:** 10.3390/biology10121235

**Published:** 2021-11-26

**Authors:** Rachel Potter, Alexis Meade, Samuel Potter, Robin L. Cooper

**Affiliations:** 1 College of Medicine, University of Kentucky, 800 Rose Street MN 150, Lexington, KY 40506, USA; rspo223@g.uky.edu (R.P.); samuel.potter@uky.edu (S.P.); 2 Department of Biology, University of Kentucky, Lexington, KY 40506, USA; Alexis.Meade@uky.edu

**Keywords:** endotoxin, insect, Na-K pump, ouabain, potassium channel

## Abstract

**Simple Summary:**

The direct action of a toxin, lipopolysaccharide, which is released from bacteria, on tissues is still not well understood. Skeletal muscle exposed to lipopolysaccharide in the larvae of the fruit fly (*Drosophila melanogaster*) causes the membrane potential to become more negative. The mechanism for this change was investigated in this study. It appears this may be due to a potassium ion leaving the cell, and this response is independent of calcium ions flowing into the cell.

**Abstract:**

The endotoxin lipopolysaccharide (LPS) from Gram-negative bacteria exerts a direct and rapid effect on tissues. While most attention is given to the downstream actions of the immune system in response to LPS, this study focuses on the direct actions of LPS on skeletal muscle in *Drosophila* *melanogaster*. It was noted in earlier studies that the membrane potential rapidly hyperpolarizes in a dose-dependent manner with exposure to LPS from *Pseudomonas aeruginosa* and *Serratia marcescens*. The response is transitory while exposed to LPS, and the effect does not appear to be due to calcium-activated potassium channels, activated nitric oxide synthase (NOS), or the opening of Cl^−^ channels. The purpose of this study was to further investigate the mechanism of the hyperpolarization of the larval *Drosophila* muscle due to exposure of LPS using several different experimental paradigms. It appears this response is unlikely related to activation of the Na-K pump or Ca^2+^ influx. The unknown activation of a K^+^ efflux could be responsible. This will be an important factor to consider in treatments of bacterial septicemia and cellular energy demands.

## 1. Introduction

Infections, which are often bacterial in origin, can impact all animals, from invertebrates to humans. When such infections are haemolymph (i.e., within invertebrates) or bloodborne, they can lead to septicaemia [1,2,3]. Around a million and half people in the USA are hospitalized each year due to septicaemia [4]. Common culprits of Gram-negative bacterial septicemia are *Escherichia coli*, *Pseudomonas aeruginosa*, and *Serratia marcescens* [5,6]. Gram-negative bacteria can trigger an immune response from the host through the secretion of endotoxins (i.e., lipopolysaccharides LPS and repeats-in-toxin RTX) [7,8]. 

The physiologic response to LPS in the host is the release of cytokines, which enhances the immune response. When excessive amounts are circulating in the blood, this may not be beneficial to the host since cytokines can cause the abnormal function of tissue, including cardiac muscle, skeletal muscle, and neural tissue [9,10,11,12]. LPS in mammals binds to a complex on tissues known as CD14/TLR4/MD2 [13]. The TLR4 protein (i.e., toll-like receptor 4) is conserved from arthropods to mammals [14]. The TLR4 receptors are primary receptors that, in part, are thought to trigger the secondary immune response of cytokine release. 

There have not yet been detailed investigations into the direct actions LPS has on cellular physiology prior to the actions of cytokines. Little is known about whether LPS directly acts on ion channels or other proteins, including receptors for hormones and neurotransmitters. The structure of LPS varies among different forms of Gram-negative bacteria. The variable O-antigen region of subtypes of LPS dictates the degree of the immune response in the host [15,16,17,18]. The direct actions of LPS itself and those stemming from secondary effects have been hard to tease out, as even direct actions of LPS may lag in time and be compounded by secondary responses. In heart tissue, it appears that LPS results in the direct action of Ca^2+^ leaking from the sarcoplasm [19]. In addition, if LPS is infused in a rodent, a decrease in heart rate occurs within a minute [20]. However, it is not established if the action occurs on heart tissue directly or the neural innervation of the heart.

Early studies using the genetic amenable model, the fruit fly (*Drosophila melanogaster*), have proven useful in helping to elucidate immune responses in mammals. This led to a Noble Prize for the researchers using *Drosophila* as well as mammals [21]. Still, *D. melanogaster* serves as a model for understanding the actions of the bacterial LPS endotoxin by detailing the initial mechanism of action and secondary cellular responses. Recently, it was demonstrated that exposure to LPS from *Pseudomonas aeruginosa* and *Serratia marcescens* rapidly hyperpolarized skeletal muscle in larval *Drosophila* and crayfish. The rapid (immediate) and transitory (minutes) response is dependent on LPS concentration and type [22,23,24,25]. In addition, the evoked and quantal postsynaptic responses in both the crayfish and *Drosophila* neuromuscular junctions (NMJs) decreased in amplitude rapidly and slowly started to increase in amplitude upon removal of LPS, which indicated that LPS had blocked the postsynaptic glutamatergic receptors. 

When the membrane was hyperpolarized, there was a larger gradient in ionic flux. This should have produced larger amplitude glutamatergic depolarizations, but the responses were eliminated or very small in amplitude. As the membrane would trend back toward membrane potential, the amplitude of the synaptic responses was still dampened. It would, however, increase with the removal of LPS. The protein sequence of glutamate subunits on larval *Drosophila* muscle partially resembles vertebrate α-amino-3-hydroxy-5-methyl-4-isoxazolepropionic acid receptors (AMPA) and kainate receptors. However, they do not share the same pharmacological profile. They do not respond well to AMPA, kainate, or N-methyl D-aspartate (NMDA) but are highly sensitive to quisqualate [26,27,28].

The two responses, hyperpolarization and glutamate receptor blockage, have not been reported prior to 2021 [22,23,24]. The mechanism behind the hyperpolarization had not yet been elucidated but was approached by investigating if the effect could be accounted for by Cl^−^ flux or tetraethylammonium (TEA)-sensitive channels (i.e., voltage-gated K^+^ or calcium-activated potassium channels). Additionally, the potential of activating NOS by LPS was addressed [25]. Experiments using TEA (20 mM) with incubation did not dampen the LPS response nor block nitric oxide synthase (NOS) with L-NAME incubation at 1 mM combined with TEA (20 mM) [24]. The hyperpolarization of LPS is not due to a flux of Cl^−^, as the equilibrium potential for Cl^−^ in larval *Drosophila* body wall muscles is more depolarized than the resting membrane potential [29,30].

The purpose of this study was to further investigate the mechanism of action by LPS on the hyperpolarization of the larval *Drosophila* muscle. Since TEA incubation did not block the response to LPS, it was assumed that it was not due to a calcium-activated potassium channel (K_(Ca)_), given that TEA is known to block this channel [31]. However, additional studies should be conducted to fully demonstrate that TEA blocks this channel in larval *Drosophila* muscle within 20 min. Another possibility is that a Na-K ATPase pump is transiently hyperactivated. Although there is not a precedent for hyperactivating the pump by a chemical mediator, it is known that the pump activity is electrogenic and is more active with depolarization. It is possible the hyperpolarization is induced by a TEA insensitive K_(Ca)_ channel, and the transit hyperpolarization is modulated by an increase in activity by the Na-K ATPase pump that slowly decreases until it returns to a homeostatic level of activity. This would allow the membrane to repolarize back to the initial membrane potential. 

## 2. Materials and Methods

Preparation and electrophysiology: Only wild-type Canton S (CS) *D. melanogaster* were used in this study. This line is an isogenic stock maintained for more than 11 years in the same laboratory and was originally obtained from Bloomington Stock Center, USA. To obtain staged larvae, the flies were held at 21 °C in a 12 h light and dark incubator before being tested, and early 3rd instars were taken from the food media. Care was taken not to use late 3rd instars due to hormonal changes and potential effects on membrane potential of the body wall muscles. All animals were maintained in standard *Drosophila* vials partially filled with a cornmeal–agar–dextrose–yeast medium.

The technique of dissecting larvae and measuring membrane potential was described in Istas et al. (2019), with the exception that all segmental nerves were transected close to the larval brain to prevent spontaneous evoked contractions induced from the CNS of the larvae. The dissections were performed by pinning the larvae down on a dish and then making a longitudinal dorsal midline cut. The internal organs were removed to expose the body wall muscle on the ventral side. A modified HL3 saline was used for physiological measures at a pH of 7.1 [32,33].

Standard saline solution (in mM): 1.0 CaCl_2_·2H_2_O, 70 NaCl, 20 MgCl_2_, 5 KCl, 10 NaHCO_3_, 5 trehalose, 115 sucrose, 25 5N, N-bis(2-hydoxyethyl)-2-aminoethanesulfonic acid (BES) at pH of 7.1 was used. The saline was modified depending on the experimental paradigm. CaCl_2_·2H_2_O was replaced by BaCl_2_ at the same concentration of 1.0 mM. CdCl_2_ or GdCl_3_ when used was added to the standard saline at 1 mM. In saline in which LPS (500 µg/mL) was used, the LPS was either added to the standard saline or to the saline containing BaCl_2_, CdCl_2_, GdCl_3_ or one containing ouabain (1 mM or 10 mM). Ouabain within 10 min was shown to substantially result in membrane depolarization in *Drosophila* larval muscle [34]. Considering that the hyperpolarization was potentially a result of a hyperactive Na-K ATPase pump, the preparations were incubated for over 10 min in ouabain (1 mM or 10 mM) prior to exposure to saline containing ouabain and LPS. LPS concentration of 500 µg/mL was used to compare with previous studies using LPS on the larval *Drosophila* muscles [22,23,24]. Commercial LPS from *S. marcescens* (*S. m*.) was dissolved in physiological saline the day of experimentation. The same concentration was used for all studies used herein in order to compare the different experimental conditions. The high concentration of LPS was also used to compare with previous studies using the muscles of frog and crayfish and the CNS of rodents [25,35]. The lethal dose for 50% of survival (LD50) in rodents for LPS from *S. marcescens* is 650 µg/mL (i.e., 6 × 10^6^ CFU colony-forming units) [36]. This was justification to use a relatively high concentration for *Drosophila* since they are likely exposed to high levels of Gram-negative bacterial strains in their native environment. This LPS may also contain some associated peptidoglycans from *S. m*. To determine if heating the saline containing LPS would dampen the effect, the saline containing LPS was boiled for 30 s, and another set of experiments underwent boiling for 5 min. The salines were brought back to room temperature prior to their use. The boiling for 5 min was conducted in an Erlenmeyer flask with a glass cover placed on the opening so condensation would return to the container; however, some vapor was lost, so the original volume was retained by adding back water to maintain salt concentrations. All chemicals were obtained from Sigma-Aldrich (St. Louis, MO, USA).

**Figure 1:**
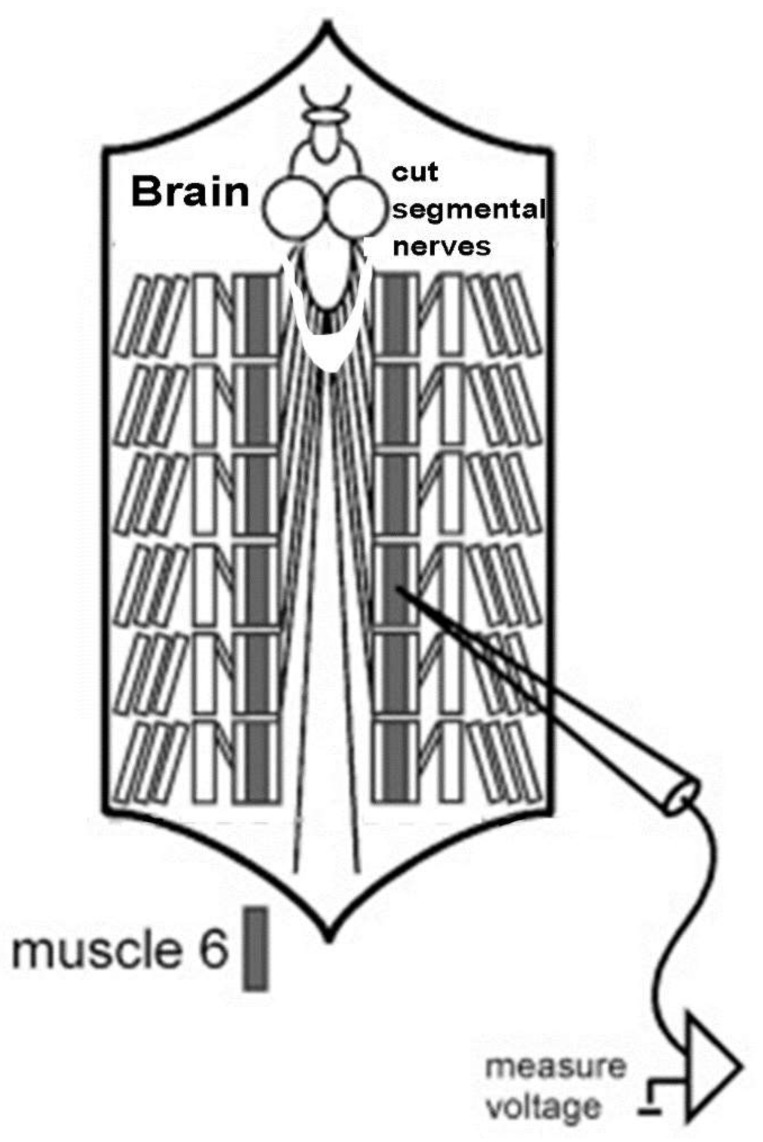
Schematic diagram of the *D. melanogaster* larva preparation for recording membrane potential in muscle 6 while changing the bathing media. The preparations consisted of pinning them with the ventral side down and holding them in place with 4 pins at the four corners. The segmental nerves were transected close to the brain. (Modified figure from Titlow and Cooper [28]).

The dissection time took 5–10 min, and it took roughly 2 min to obtain an intracellular recording from a muscle fiber. Intracellular microelectrode recordings were all performed in muscle fiber 6 (m6) in segments 3 or 4. (Figure 1). Glass microelectrodes were filled with 3 M KC) and an AxoClamp-2 B amplifier (Molecular Devices, LLC, 1311 Orleans Drive, Sunnyvale, CA, USA) was used. The signals were recorded via a PowerLab/4 s interface (ADI Instruments, Colorado Springs, CO, USA) at 20 kHz. A ground wire was placed within a 1% agar plug. The agar was made in standard saline without Ca^2+^ and was placed in plastic Eppendorf pipette tips with the tip cut back at a diagonal and held in place with surgical wax. This was to ensure that there was no electrical potential offset by the changes in fluid level on the ground wire when solutions were exchanged. 

Experimental design: The membrane potential of m6 was measured in standard saline for at least one minute before the bath was exchanged to a saline with other compounds or altered salt concentrations, as shown and detailed in the Section 3. The total volume of the chamber was less than 0.5 mL and was generally exchanged three times with each bathing solution except for the addition of LPS. LPS saline was readily exchanged over the dissected preparations during the recording of membrane potential. The change in membrane potential is so rapid with the LPS addition that only one exchange in the bathing media was required. 

The paradigms used are presented in Table 1 and highlighted with each representative trace of the changes in the membrane potentials for the paradigm in the Section 3.

Each condition shown was when membrane potential was monitored. Exchanges in saline bathing media are shown with a slash line. Standard saline is HL3 saline (in mM): 1.0 CaCl_2_·2H_2_O, 70 NaCl, 20 MgCl_2_, 5 KCl, 10 NaHCO_3_, 5 trehalose, 115 sucrose, 25 5N, N-bis(2-hydoxyethyl)-2-aminoethanesulfonic acid (BES) at pH of 7.1.

Statistical analysis: Statistical analysis was performed as a sign pairwise test for changes in membrane potential. Since some data sets were used to examine a direct change in the membrane potential, the non-parametric sign test was used. A significant difference is considered as *p* < 0.05.

## 3. Results

**Figure 2:**
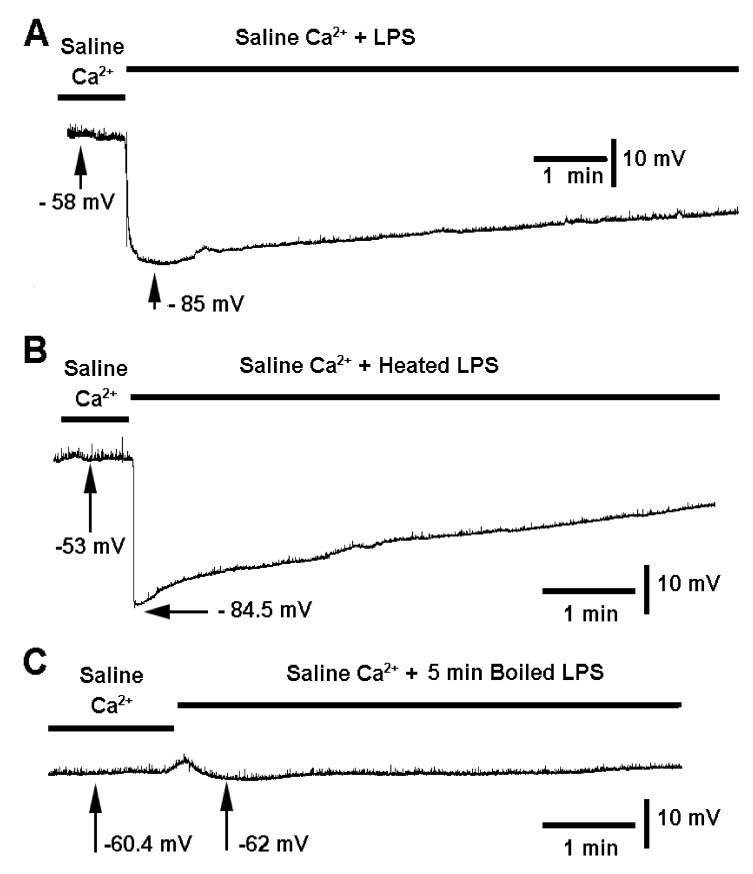
The effect of lipopolysaccharide (LPS) from *Serratia marcescens* (500 µg/mL) on membrane potential of larval *Drosophila* skeletal muscle. (**A**) LPS dissolved in standard saline produces rapid hyperpolarization. (**B**) The response on the membrane potential is not different if the LPS was previously boiled for 30 s. (**C**) There is mild hyperpolarization with saline containing LPS, which was boiled for 5 min and corrected for vapor loss. These responses were of the same trends in six preparations for each paradigm (*n* = 6; *p* < 0.05, sign test, non-parametric).

In this investigation, the focus is on the membrane potential of the muscle fibers and not on synaptic transmission. To illustrate the effect of LPS in saline containing Ca^2+^ on membrane potential, a representative trace with a prolonged exposure is shown (Figure 2A). The membrane potential rapidly hyperpolarizes and gradually depolarizes until returning to the initial potential. Every preparation varies in the rate of repolarization. Some will repolarize more rapidly, but all show a rapid hyperpolarization to LPS exposure (*n* = 6; *p* < 0.05, sign test, non-parametric). To determine if heating the saline containing LPS would dampen the effect, the saline containing LPS was boiled for 30 s and brought back to room temperature prior to using. The effects were similar to the conditions without boiling. Each preparation showed the rapid hyperpolarization (Figure 2B; *n* = 6; *p* < 0.05, sign test, non-parametric). However, when the saline containing LPS was vigorously boiled for 5 min and corrected for vapor loss by adding water back, the membrane did not produce a significant change in membrane potential (*n* = 9; mean of −2.311 mV +/− SEM of 0.52). A representative preparation of the effect with vigorously boiled saline containing LPS is illustrated in Figure 2C.

**Figure 3:**
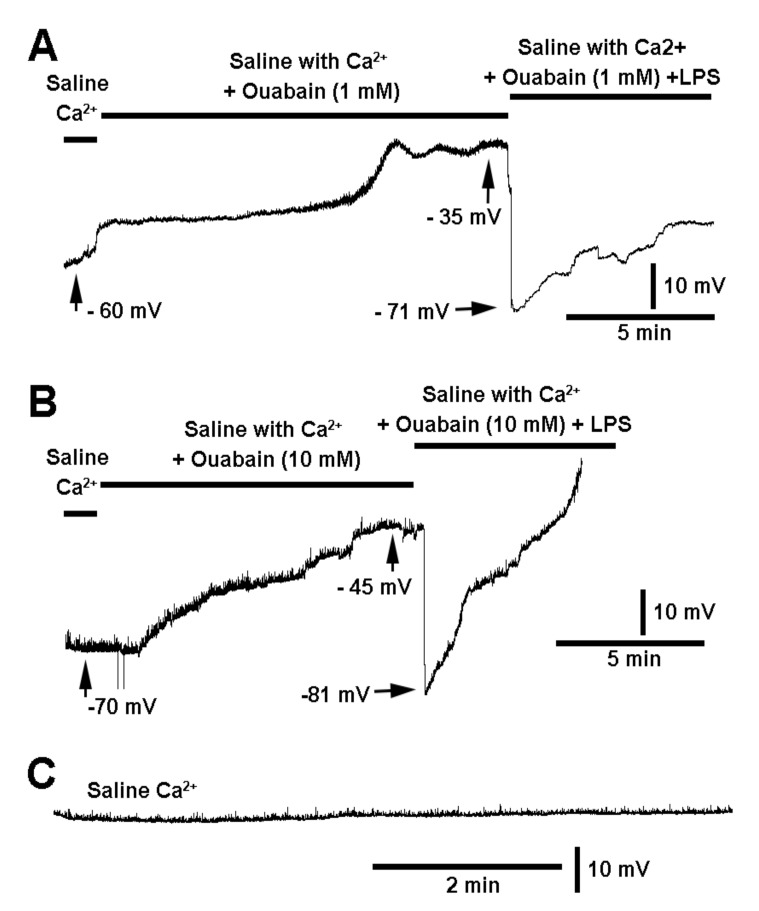
The effect of lipopolysaccharide (LPS) when the Na-K ATPase pump is inhibited by ouabain. Ouabain exposure to the larval *Drosophila* muscle slowly depolarizes the membrane potential to the same degree for 1 mM (**A**) and 10 mM (**B**) over a 10 min period. Upon exposure to LPS, with preincubation of ouabain, there was still a hyperpolarization of the membrane (*n* = 6 for each concentration of ouabain; *p* < 0.05, sign test, non-parametric). (**C**) The membrane is fairly stable over a period of time, indicating ouabain exposure results in depolarization of the membrane potential.

The saline used in this paradigm did contain Ca^2+^, so as not to confuse the effect of the depolarization due to ouabain with depolarization induced by low [Ca^2+^]_o_. Each preparation showed a depolarization over time during incubation with ouabain (*n* = 12; *p* < 0.05, sign test, non-parametric) and the preparations in which LPS was used in combination with ouabain hyperpolarized (Figure 3; *n* = 6; *p* < 0.05, sign test, non-parametric). The 1 mM and 10 mM exposure to ouabain depolarized the membrane to the same extent, suggesting 1 mM is sufficient to block the pump. With either concentration of ouabain, the membrane still rapidly hyperpolarized with exposure to LPS in saline containing the same concentration of ouabain during the 10 min pre-exposure. (Figure 3A,B). The membrane potential is fairly stable when there is no exposure to ouabain or LPS (Figure 3C). 

**Figure 4:**
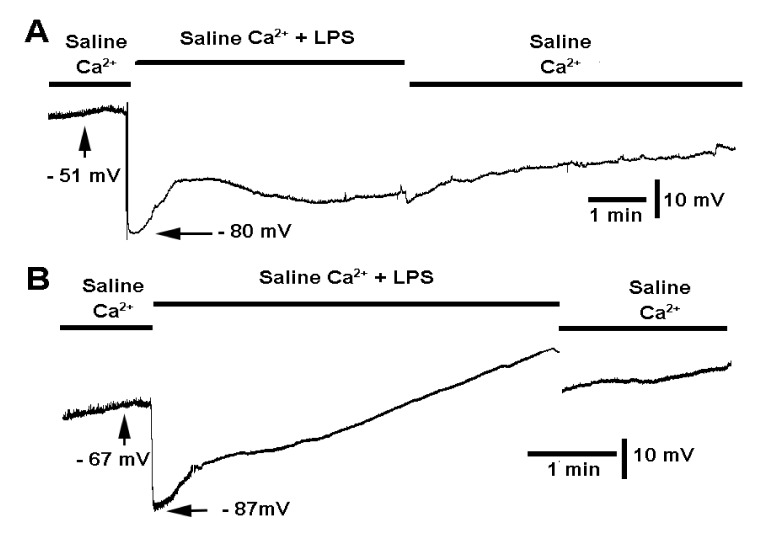
The effect of lipopolysaccharide (LPS) from *Serratia marcescens* (500 µg/mL) in standard saline and the effect of exchanging out for normal saline. (**A**) Some preparations do not appear to rapidly recover upon exchanging fresh saline not containing LPS. (**B**) In some cases, membrane potential will recover rapidly and will remove the block of the postsynaptic quantal responses.

In previous studies, while examining the effects of LPS on evoked and spontaneous quantal synaptic transmission, there was variation among preparations in the ability to wash out the effects of LPS. In the case that inducing evoked transmission had additional effects in altering the membrane potential recovery, in this study, the nerve was not stimulated; however, there was still substantial variation in recovery after the removal of LPS among preparations, as shown in Figure 4A,B. The wash out of saline containing LPS was performed by flushing the saline bath with three exchanges of fresh saline. Care is required as not to dislodge the intracellular electrode. 

**Figure 5:**
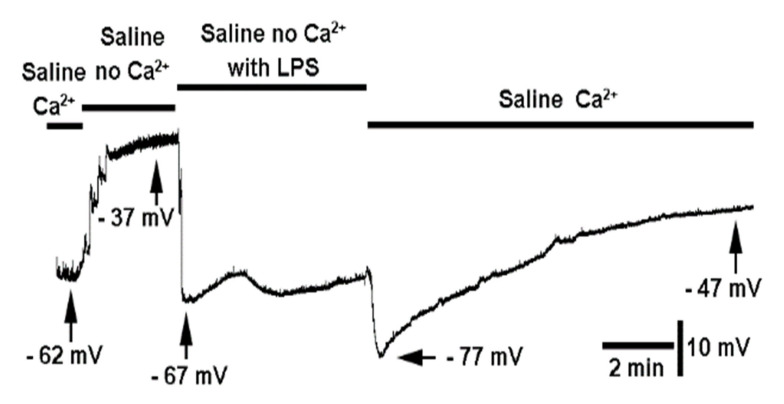
The effect on membrane potential of saline free of Ca^2+^ and of LPS in Ca2+-free saline. Saline not containing Ca^2+^ results in depolarization of the membrane, and subsequent exposure to saline without Ca^2+^ while containing LPS produces a rapid hyperpolarization. Upon removal of the saline without Ca^2+^ and LPS to one with Ca^2+^ and no LPS produces a secondary hyperpolarization (*n* = 6; *p* < 0.05, sign test, non-parametric).

To examine the potential of the influx of Ca^2+^ ions triggering a rapid activation of a K(Ca) current to account for the hyperpolarization, a Ca^2+^-free saline was used. Preparations were bathed in the saline for 2 min prior to exposure to LPS. The initial saline was flushed three times with the saline not containing any added Ca^2+^. This was followed by the saline without Ca^2+^ but with LPS. In every preparation, there was depolarization as a result of switching the saline to one without Ca^2+^ added. However, the addition of LPS with saline without Ca^2+^ still produced a pronounced hyperpolarization in the preparations (Figure 5; *n* = 6; *p* < 0.05, sign test, non-parametric). Upon switching the saline back to one containing Ca^2+^ without LPS, there was still a slight hyperpolarization. This response could be related to compensation for the saline without Ca^2+^ or an effect of LPS still present on the membrane and responding once Ca^2+^ was added. 

**Figure 6:**
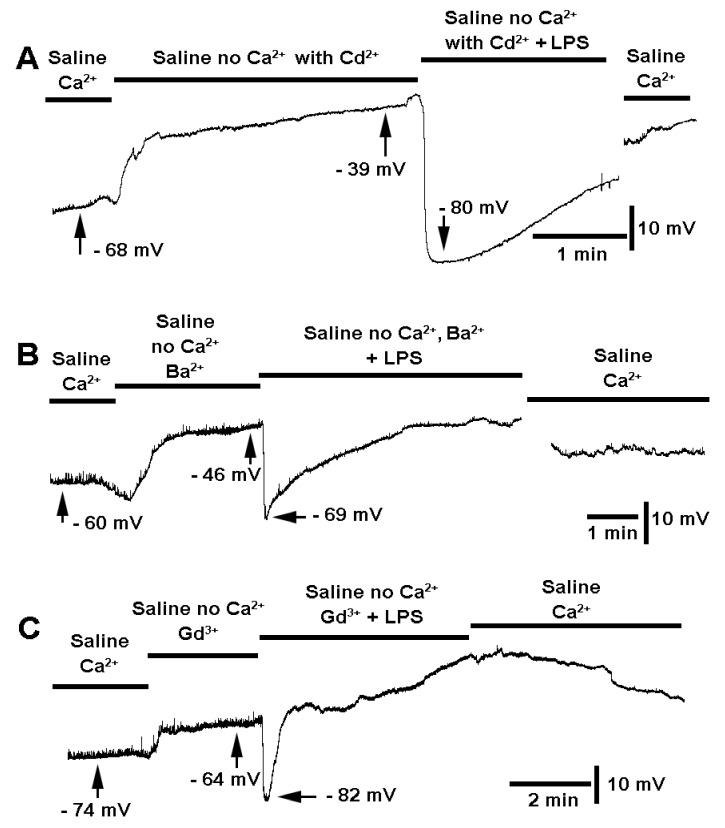
The effect on membrane potential of saline with Ca^2+^ substituted for Cd^2+^, Ba^2+^, or Gd^3+^ and the effect of LPS on the membrane potential in these modified solutions. The substitutions were made for other ionic compounds at the same concentration of 1 mM. With Cd^2+^(**A**), Ba^2+^(**B**) or Gd^3+^(**C**) substituted for Ca^2+^ caused the membrane to depolarize. In addition, each modified saline containing LPS rapidly produced a hyperpolarization (each saline with *n* = 6; *p* < 0.05, sign test, non-parametric).

The skeletal muscle of larval *Drosophila* conducts electrical depolarization by voltage-gated Na^+^ and Ca^2+^ channels on the plasma membrane. Since Ca^2+^ entry can potentially activate K(Ca) channels, Cd^2+^ was substituted for Ca^2+^ in the saline prior to LPS exposure. The incubation in Ca2+-free saline containing Cd^2+^ would block all voltage-gated Ca^2+^ channels on the plasma membrane. The Ca2+-free saline still produced depolarization of the membrane, indicating Cd^2+^ does not replace the effect of Ca^2+^ in this regard. This effect was present in all preparations examined (Figure 6; *n* = 6; *p* < 0.05, sign test, non-parametric). The additional exposure to LPS in saline without Ca^2+^ but containing Cd^2+^ still produced a rapid hyperpolarization (Figure 6A; *n* = 6; *p* < 0.05, sign test, non-parametric). Initially, the effect of Cd^2+^ was examined in standard saline containing Ca^2+^, but since the effect of exposure to LPS was still prominent in a hyperpolarization (data not shown; *n* = 6; *p* < 0.05, sign test, non-parametric) a saline without Ca^2+^ added was examined, assuming that no potential Ca^2+^ would enter the muscle. An additional approach to potentially block Ca^2+^ influx and not activate K(Ca) channels was examined using Ba^2+^ and gadolinium chloride (Gd^3+^). The same trends were observed for [Ca^2+^]_o_ that was substituted for the same [Ba^2+^]_o_ or [Gd^3+^]_o._ In each preparation, the membrane was depolarized (Figure 6B,C; each *n* = 6; *p* < 0.05, sign test, non-parametric). Additionally, LPS exposure in saline with the same [Ca^2+^]_o_ substituted for [Ba^2+^]_o_ or [Gd^3+^]_o_ still showed rapid hyperpolarization (Figure 6B,C; each *n* = 6; *p* < 0.05, sign test, non-parametric).

## 4. Discussion

This study was designed to address the mechanism underlying the hyperpolarization of the membrane potential induced by exposure to LPS. It was demonstrated that even boiling saline containing LPS for 30 s still resulted in hyperpolarization and blocking of the glutamate receptors. However, vigorously boiled saline with LPS showed little effect on the membrane potential, likely due to some denaturing of LPS. The potential of a rapid activation of the Na-K ATPase pump induced by LPS was ruled out due to LPS still producing hyperpolarization when the pump was blocked. Even with a prior incubation of ouabain greater than 10 min and the use of a high concentration of ouabain (10 mM), LPS still produced a hyperpolarization. Since muscle contraction is observed with exposure to LPS while the muscle hyperpolarizes, it was assumed the Ca^2+^ influx was induced by LPS. In addition, some preparations were not utilized due to the muscle contracting when exposed to LPS. As noted in earlier studies, sometimes there would be a burst of vesicular fusion events with LPS exposure, which could induce the muscle to contract [22,23,24]. As illustrated in the traces shown in Figure 4A,B and others presented in this study, the spontaneous quantal events in the initial recording generally vanish with LPS exposure due to the partial blocking of the postsynaptic glutamate receptors. In some cases, the minis will return with washout back to standard saline. This phenomenon was detailed in earlier reports [22,23,24].

Given that the larval *Drosophila* body wall muscle is dependent on voltage-gated Ca^2+^ channels in the plasma membrane for depolarization-induced muscle contraction, similarly to mammalian cardiac muscle, it seemed feasible that an influx of Ca^2+^ was induced by LPS. This is confirmed by an earlier report that demonstrated the membrane potential depolarizes in a concentration-dependent manner when [Ca^2+^]_o_ is reduced [37]. It was shown when there was depolarization as a result of switching the saline to one without Ca^2+^ added. It has been suggested that the depolarization of the membrane potential due to reduced [Ca^2+^]_o_ and the hyperpolarization due to raising [Ca^2+^]_o_ is likely induced by calcium-activated potassium channels K(Ca) [37]. So, to address if the mechanism behind the hyperpolarization induced by LPS is due to a Ca^2+^ influx rapidly activating a K(Ca), the manipulation of Ca^2+^ influx and ionic substitution was implemented. The results from substituting [Ca^2+^]_o_ for [Ba^2+^]_o_, [Cd^2+^]_o_, and [Gd^3+^]_o_ would indicate that there is likely a LPS-activated type of K^+^ channel or a K(Ca) current not blocked by these ionic substitutions responsible for the hyperpolarization.

It was surprising that rapidly boiling the saline containing LPS for only a few seconds did not dampen the effect of LPS by hyperpolarizing the membrane potential, but a longer boiling time of 5 min blocked the effect of LPS. The 5 min boiling time for saline did result in some evaporation, so added water was required as not to alter concentration of the salts. Considering the possibility of the Na-K ATPase pump being rapidly hyperactivated, the pre-incubation of 10 min with 1 mM or 10 mM ouabain, still with a pronounced hyperpolarization when exposed to LPS combined with ouabain, would indicate the pump is not likely an explanation for the effect of LPS. It is possible ouabain is not that effective at inhibiting the body wall muscle isoform of the Na-K ATPase pump in *Drosophila*. However, the membrane potential did gradually depolarize with exposure to ouabain over 10 min, which was also shown to occur in prior studies [34]. Thus, it is assumed the Na-K ATPase pump is blocked in this larval *Drosophila* muscle preparation.

The addition of Ba^2+^ (1 mM) to the standard saline with 2 min pre-exposure before LPS did not result in a loss of hyperpolarization or muscle contraction. It is likely that the [Ba^2+^]_o_ was not a high enough concentration to reduce the effect of Ca^2+^ on K(Ca) or induction of muscle contraction. In saline substituting [Ca^2+^]_o_ for [Ba^2+^]_o_, depolarization of the membrane potential still occurred. So, Ba^2+^ does not appear to block the effect of the membrane depolarization by reduced [Ca^2+^]_o_. The addition of LPS in saline with Ba^2+^ substituted for Ca^2+^ greatly reduced the hyperpolarization. Thus, it is feasible that the depolarization of muscle with lowered [Ca^2+^]_o_ is due to an effect on screening of the membrane and proteins by Ca^2+^ [38,39] and that the hyperpolarization by LPS was induced by a potential K(Ca) current that is not inhibited by Ba^2+^.

To further investigate whether the depolarization with reduced [Ca^2+^]_o_ was due to a depressed K(Ca) current or a screening of membrane charge, [Ca^2+^]_o_ was substituted for [Cd^2+^]_o_. Cd^2+^ normally blocks voltage-gated Ca^2+^ channels. Interestingly, the membrane still depolarized with [Ca^2+^]_o_ (1 mM) substituted for [Cd^2+^]_o_ (1 mM); furthermore, the membrane was still hyperpolarized when exposed to LPS. Thus, this would suggest that if K(Ca) current is responsible for the LPS-induced hyperpolarization, Cd^2+^ is activating a K(Ca) current. This is feasible given that cadmium or strontium can activate K(Ca) channels in neurons of mollusks [40,41]. In addition, divalent cations such as Cd^2+^ can activate small- (SK) and large-conductance (BK) channels in mouse neuroblastoma cells [42]. Interestingly, K(Ca) is also known to be intrinsically voltage dependent [43]. 

The use of [Gd^3+^]_o_ as a substitute for [Ca^2+^]_o_ still resulted in depolarization of the membrane, and the addition LPS still produced hyperpolarization. This would indicate that the depolarization is induced by a Ca^2+^ mechanism that cannot be substituted by Gd^3+^. The LPS hyperpolarization may be connected to a similar mechanism for all ionic substitutions. The LPS-induced hyperpolarization was not blocked in any of the ionic substitution experiments. Both phenomena of membrane potential changes indicate that K+ channels or K(Ca) channels could account for the effects. Some studies have used a substitution of Ca^2+^ for Gd^3+^ to block K(Ca) effects in smooth muscle [44], but this does not appear to be a common experimental approach to assess K(Ca) currents. Gd^3+^ is toxic at high concentrations, as it is known to block presynaptic Ca^2+^ channels in nerves and Ca^2+^ channels in cardiac tissue [45,46,47,48]. 

Since the muscle can still contract when Cd^2+^ substitutes for Ca^2+^, an internal release of Ca^2+^ from a sarcoplasmic reticulum (SER) is suggested. However, larval *Drosophila* skeletal muscle does not have a robust SER. It would appear that Cd^2+^ can also substitute for Ca^2+^ in binding to troponin to induce skeletal muscle contraction, as is reported in other studies [49,50]. So, this would also indicate that in our studies, Cd^2+^ was able to move into the cytoplasm of the muscle to come into contact with troponin.

Other possible scenarios in the mechanism behind the LPS-induced hyperpolarization were considered, such as activating a Cl^−^ channel. Considering that the chloride equilibrium potential (−40 to −20 mV in Cl^−^ loaded fibers) is above resting membrane potential in the body wall muscle of *Drosophila* larvae, this possibility was not an option [29,30]. Muscles with a chloride equilibrium potential above the resting membrane potential are not uncommon, considering that body wall muscle in moths also has more depolarized chloride equilibrium potential than resting membrane potential [51]. TEA incubation for 10 min in prior studies using larval *Drosophila* muscle did not block the LPS-induced hyperpolarization [25]; however, those studies were conducted in the presence of standard saline, and TEA might not have permeated the muscle cell to block K(Ca) from the cytoplasmic side. The possibility of NOS in muscle being activated by LPS was considered earlier and addressed by incubation in L-NAME to inhibit NOS, which did not block the responses by LPS [25].

How the membrane can become so rapidly hyperpolarized to values such as −80 mV by LPS exposure needs to be addressed by ionic driving gradients to account for the change. Early studies in adult *Drosophila* muscle and moth muscle indicated that the muscle potential for K^+^ may exceed −90 mV [52,53]. This greater negative potential than that found in the estimated K^+^ reversal potential of around −50 to −60 mV was rationalized by pointing out the more active metabolic processes, as compared to those taken into consideration in the experimental paradigms to measure K^+^ reversal potential [34,51,53,54]. However, studies on stick insects indicated no significant difference between the reversal potential of the delayed K^+^ current and the resting potential [55]. This illustrates there are differences depending on the insect examined in these K^+^ current measures, and not all insects will show similar responses to LPS exposure to the body wall muscles. In addition, the larval and adult forms of body wall muscles may likely differ in equilibrium potential for K^+^. 

Since the dissections and time taken to measure membrane potentials are relatively rapid, it makes it unlikely that active metabolic processes can explain the differences in measured K^+^ equilibrium potential and measured K^+^ reversal potential. The equilibrium potential for K^+^ is very negative. The presence of a Na^+^ leak, a constituently active K(Ca) current, or even a Cl^−^ leak in an aggerate of fluxes could maintain the membrane potential at rest more depolarized than the equilibrium potential for K^+^. It is likely these other currents were not blocked or experimentally considered when determining the K^+^ reversal potential as a measure of the K^+^ equilibrium potential in earlier studies [56].

It is most likely that LPS exposure activates a K^+^ current either directly or through K(Ca) channels to result in transient hyperpolarization. It is feasible that the hyperpolarization slowly decreases as the membrane repolarizes due to the activity of the Na-K ATPase pump. Since the pump is electrogenic and not as active with hyperpolarization as depolarization, this homeostatic drive is slower with hyperpolarization. However, the prolonged exposure to LPS will result in a depolarized state, which is possibly due to plasma membrane break downing as the resting membrane potential is lost. This cell degradation might be induced by an excess of Ca^2+^ or Na^+^ loading over time.

## 5. Future Directions

Additional pharmacological approaches for examination in larval *Drosophila*, as used in mammalian tissues, can be tested to target K^+^ channels and pumps. However, the agents need to be confirmed to work on *Drosophila* isoforms of the proteins [57]. It would be of interest to know if similar phenomena occur by analogous mechanisms in the skeletal muscles of other invertebrates. This approach we are now addressing in the skeletal muscle of crustaceans. The robust nature of crustacean muscle preparations and ease in maintaining intracellular recordings while exchanging the bathing media allows them to be good experimental models for examining the effects of LPS. There is a robust transient hyperpolarization with exposure to LPS in crayfish skeletal muscle that has yet to be fully investigated [22,23,58,59]. In addition, the actions of LPS on neuronal properties in crustacean models can be addressed, as motor axons are generally large enough to obtain intracellular recordings of identified neurons and inject substances to block channels and second messenger cascades [60,61].

## Figures and Tables

**Table 1:** Paradigms used for assessment in the effects by LPS exposure.

Paradigm Type Conditions Used
Paradigm 1 Std. saline/Std. saline + LPS/Std. saline
Paradigm 2 Std. saline/Std. saline + boiled LPS/Std. saline
Paradigm 3 Std. saline/Std. saline + ouabain/Std. saline + ouabain + LPS/Std. saline
Paradigm 4 Std. saline/No Ca^2+^ saline/No Ca^2+^ saline + LPS/Std. saline
Paradigm 5 Std. saline/No Ca^2+^ saline + Ba^2+^/No Ca^2+^ saline + Ba^2+^ + LPS/Std. saline
Paradigm 6 Std. saline/Std. saline + Cd^2+^/Std. saline + Cd^2+^ + LPS/Std. saline
Paradigm 7 Std. saline/No Ca^2+^ saline + Cd^2+^/No Ca^2+^ saline + Cd^2+^ + LPS/Std. saline
Paradigm 8 Std. saline/No Ca^2+^ saline + Gd^3+^/No Ca^2+^ saline+ Gd^3+^ + LPS/Std. saline

## Data Availability

All data are available in manuscript and available upon request.
